# A monoclonal antibody targeting spore wall protein 1 inhibits the proliferation of *Nosema bombycis* in *Bombyx mori*

**DOI:** 10.1128/spectrum.00681-23

**Published:** 2023-10-09

**Authors:** Bin Yu, Rong Zheng, Maofei Bian, Ting Liu, Kun Lu, Jialing Bao, Guoqing Pan, Zeyang Zhou, Chunfeng Li

**Affiliations:** 1 State Key Laboratory of Silkworm Genome Biology, Southwest University, Chongqing, China; 2 Chongqing Key Laboratory of Microsporidia Infection and Prevention, Southwest University, Chongqing, China; 3 Chongqing Three Gorges Medical College, Chongqing, China; 4 College of Life Sciences, Chongqing Normal University, Chongqing, China; Broad Institute, Cambridge, Massachusetts, USA

**Keywords:** microsporidia, *Nosema bombycis*, *Bombyx mori*, monoclonal antibodies, transgene

## Abstract

Microsporidia are obligate intracellular single-cell eukaryotic parasites that can infect almost all kinds of animals and cause microsporidiosis. However, there are a few achievements of microsporidiosis treatments. *Nosema bombycis* could cause a microsporidiosis condition, pébrine, in the silkworm. Treatment and prevention of pébrine are still the research hotspots in sericulture. In this study, *N. bombycis* was treated with K_2_CO_3_, the spore walls were removed by centrifugation, and then, the alkali-soluble germination proteins were used as antigens to develop monoclonal antibodies (mAbs). Three mAbs were successfully screened and one mAb named G9 showed the highest titer among these monoclonal antibodies after enzyme-linked-immunosorbent serologic assay (ELISA). Mass spectrometry analysis confirmed that the mAb G9 could recognize the *N. bombycis* spore wall protein 1. Furthermore, the heavy chain and light chain sequences of the G9 monoclonal antibody were cloned, respectively. The vectors that expressing the intact antibodies and the single-chain variable fragments (scFvs) of G9 were constructed, and then, these vectors were used to develop the transgenic silkworm cell lines or transgenic silkworms. The inhibitory effects against *N. bombycis* were evaluated by the count of microsporidia and qPCR. The scFvs showed better effect on blocking the proliferation of *N. bombycis* than the intact antibody, and the scFv without the secretory signal peptide was more effective than that with signal peptide. Our study has provided novel strategies for microsporidiosis control and the essential groundwork for the future development of *N. bombycis-*resistant transgenic silkworms.

**IMPORTANCE:**

There are a few reports on the resistance of microsporidia, including *Nosema bombycis*. Here, the alkali-soluble germination proteins of *N. bombycis* were used as immunogens to prepare a monoclonal antibody, and its single-chain variable fragments effectively blocked microsporidia infection. Our study has provided novel strategies for microsporidiosis control and demonstrated a useful method for the potential treatment of other microsporidia diseases.

## INTRODUCTION

Microsporidia are obligate intracellular single-cell eukaryotes that parasitize vertebrates and invertebrates (1, 2) and cause multiple host diseases. In insects, *Nosema bombycis* can cause pébrine (3), *Nosema ceranae* leads to honeybee colony collapse (4, 5), and *Antonospora locustae* (formerly *Nosema locustae*, a pathogen of locusts and grasshoppers) was developed as a biological control agent (6, 7). In aquaculture, microsporidia can cause the mortality of fish, prawn, and crab (8, 9). Microsporidia infections bring about mammalian livestock diseases (10, 11), and some microsporidia can also cause human diseases (12, 13). A total of 200 genera and 1,500 species of microsporidia have been reported so far (14), but there are a few reports on microsporidiosis treatment. At the present time, albendazole and fumagillin are the most effective compounds against *Encephalitozoon intestinalis* and *Enterocytozoon bieneusi*, respectively (15). However, these drugs are only effective against a single or several microsporidia species. There is a lack of treatment regimens for most microsporidia. Therefore, new microsporidia prevention and treatment techniques are needed to suppress microsporidia infection and improve disease treatment (16).

*N. bombycis* was the first identified microsporidia. Like other microsporidia, *N. bombycis* forms mature dormant spores to ensure survival under environmental stress. These dormant microsporidia have a thick spore wall composed of proteins and chitin (17, 18) that protects against unfavorable external environments. The polar tube will extrude from cells when spores encounter external stimuli and release cytoplasm to complete infection (19). At present, the control of *N. bombycis* mainly relies on disinfectants such as bleaching powder and formaldehyde to help prevent silkworm from infection (20). However, these methods have little therapeutic effects on *N. bombycis*-infected silkworms.

The monoclonal antibody (mAb) has been widely used for prevention as well as for treatments. The effectiveness relies on multiple mechanisms such as direct blocking of pathogen entry (neutralization), mAb-mediated effector functions, or indirect blocking of pathogen entry (21 – 25). For instances, mAb against the spike protein of SARS-CoV-2 for the prevention of COVID-19 achieved good prevention/treatment effects and was approved by FDA for emergency usage (26). Single-chain variable fragments (scFvs) were developed to block the transmission of malaria-causing *Plasmodium falciparum* in mosquitoes. The expression of scFvs targets essential surface proteins and secretory proteins of *P. falciparum* in mosquitoes, which significantly decreases adult infection levels (27 – 30). Similar to *P. falciparum*, *N. bombycis* is also an obligate intracellular parasite. Therefore, using monoclonal antibodies to block *N. bombycis* infection in silkworms may also be a feasible and effective strategy for pébrine prevention and treatment.

In this study, the *N. bombycis* spore was treated with K_2_CO_3_ and removed the spore walls. The mixture of spore germination fluid was used as immunogen to prepare monoclonal antibodies. The expression of antibodies could effectively inhibit the proliferation of *N. bombycis* in silkworm. Our study has provided novel strategy for pebrine prevention and control.

## RESULTS

### Preparation of monoclonal antibodies

**Fig 1:**
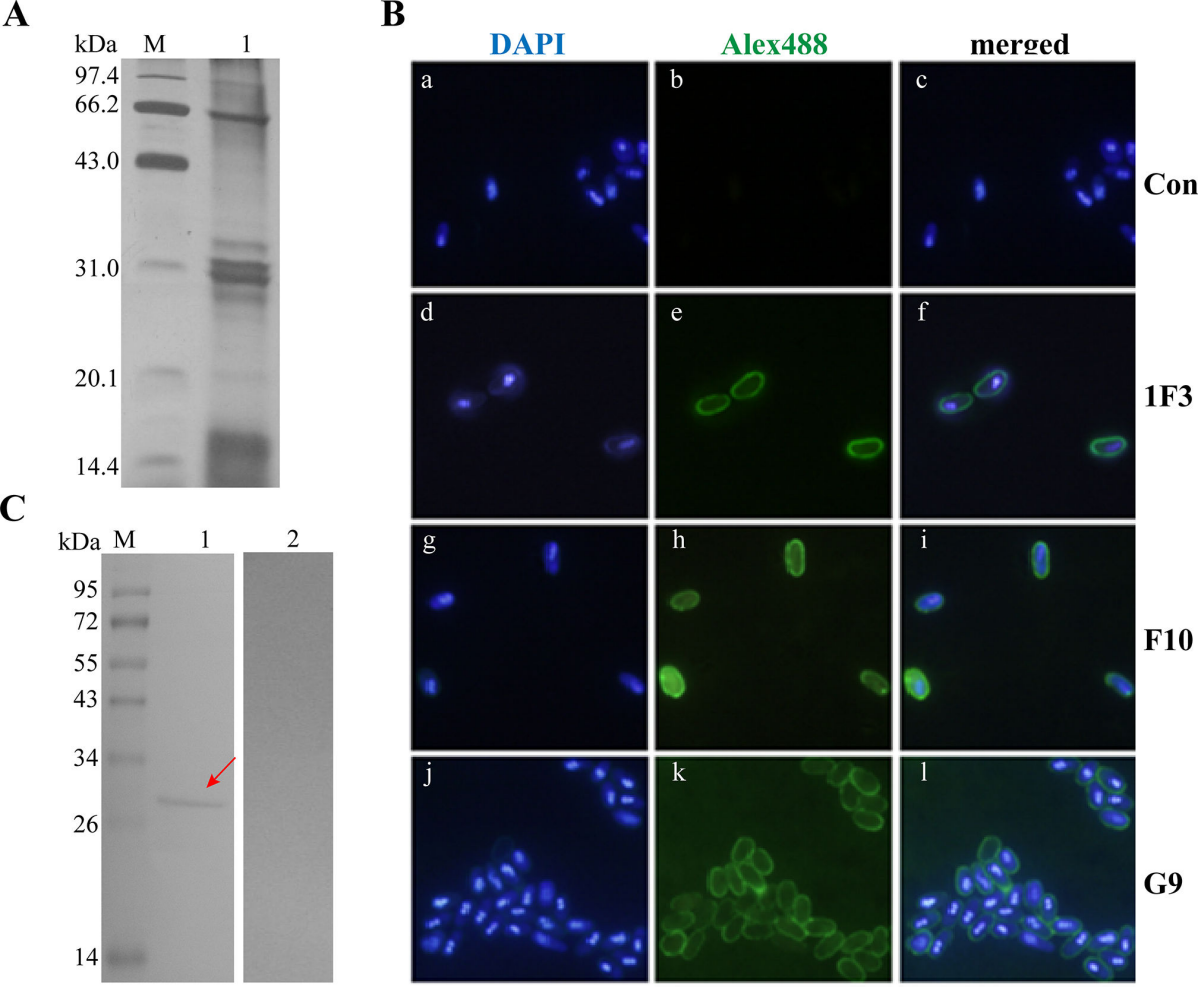
Preparation of monoclonal antibody against alkali-soluble germination protein of *N. bombycis*. (A) The SDS-PAGE showed abundant germination liquid protein was enriched by removing spore shell after germination. (B) Subcellular localization of the target protein recognized by mAb through immunofluorescence assay (IFA). The blue fluorescent signal represents the nucleus labeled with DAPI (a, d, g, j). The green fluorescent signal was observed on the surface of *N. bombycis* incubated with mAb 1F3 (e), F10 (h), and G9 (k), but not with negative serum (b). (C) Germination liquid proteins were used to detect specificity of mAb G9 by Western blotting. A hybridization band (red arrow) was found in mAb G9 (line 1) but not in negative serum (line 2).

The complex interactions between *N. bombycis* and hosts begin in the midgut. The midgut lumen contents of silkworms, especially the feeding larvae, are strongly alkaline (31). Under the alkaline environment, mature spore of *N. bombycis* will germinate and infect host cells. In order to prepare proteins enriched during spore germination, we incubated *N. bombycis* spores with K_2_CO_3_ to induce spore germination (Fig. S1). The alkali-soluble germination proteins mixture (*N. bombycis* was treated with K_2_CO_3_ and the spore walls were removed by centrifugation) was subsequently used as antigens for mouse immunization (Fig. 1A). Splenocytes were isolated from immunized mice and fused with SP2/0 cells. After three rounds of screening, three mAbs (1F3, F10, and G9) were obtained.

We further validated the specificity of the three mAbs by immunofluorescence assay (IFA), which showed that strong fluorescence signals were present on spore wall in the groups incubated with the mAbs but not in control group (Fig. 1B). ELISA analysis showed that the titer of G9 was the highest among the three mAbs (Table S1). Therefore, G9 mAb was screened for subsequent experiments. We next confirmed that the G9 mAb antigen specificity for recognizing germination fluid proteins by Western blotting. As shown in Fig. 1A and C, specific band of approximately 30 kDa was recognized by G9 mAb but not by control serum. This result indicated that G9 mAb can specifically recognize proteins in spore wall. In summary, our results showed that a mAb (G9) recognizing proteins on spore wall has been successfully developed by using alkali-soluble germination proteins as antigen.

### Identification of target protein recognized by G9 mAb

**Fig 2:**
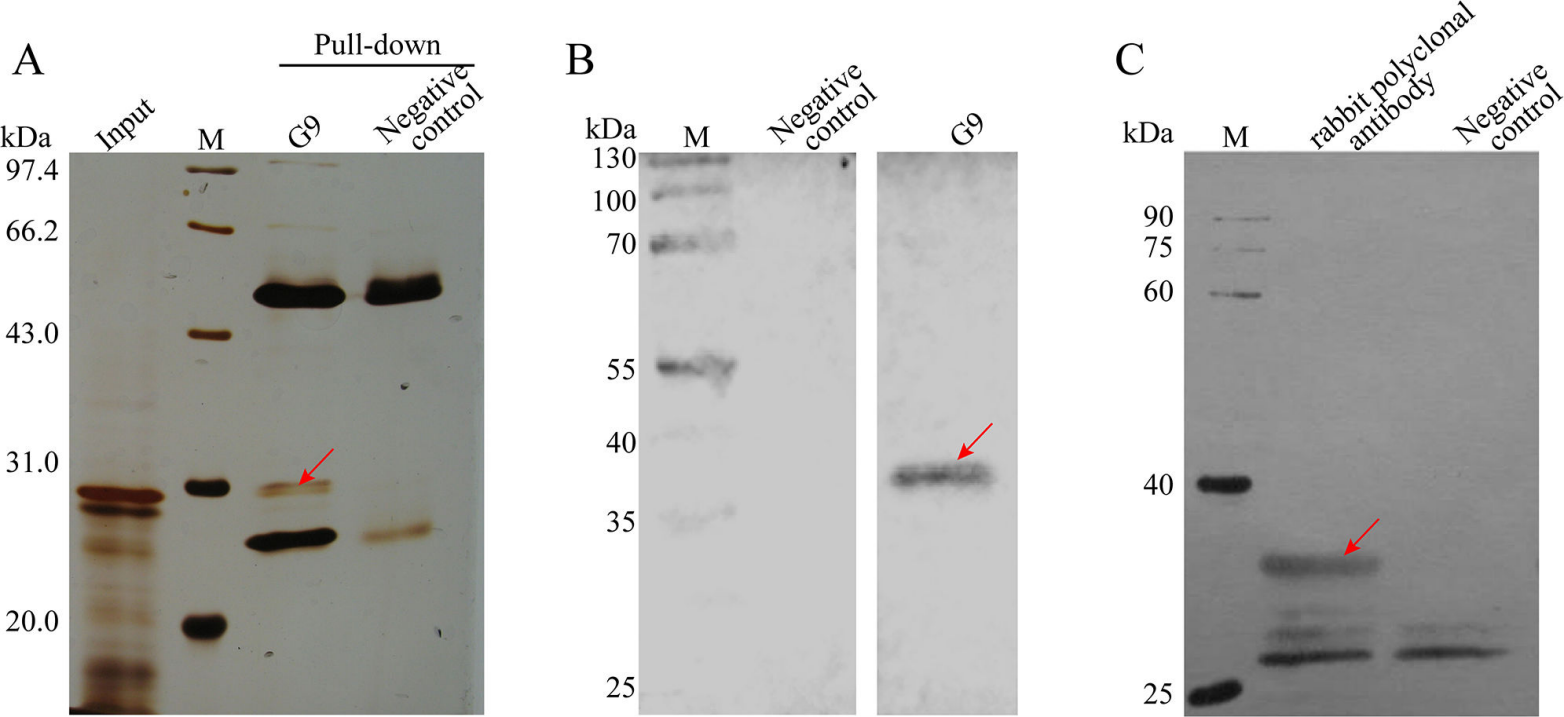
Identification of the target protein recognized by mAb G9. (A) The pull-down assay was used to identify the target protein of mAb G9. There is an obvious difference band (the red arrow) between G9 and negative serum. (B) The recombinant SWP1 (rSWP1) of *N. bombycis* was detected with mAb G9 by Western blotting, while negative serum was used as a negative control. (C) The rabbit polyclonal antibody against rSWP1 was prepared, and the native SWP1 was detected in the sample of G9 pull-down by rabbit polyclonal antibody, while the negative serum was used as negative control.

To identify the specific protein recognized by G9 mAb, immunoprecipitation was performed using G9 mAb-coated protein A + G beads and naïve murine serum was included as a negative control. As shown in Fig. 2A, a band of approximately 30 kDa was unique for G9 mAb immunoprecipitation in SDS-PAGE gel but was absent when naïve serum was used (Fig. 2A). The specific band of about 30 kDa was cut for mass spectrometry analysis, and the results showed that SWP1 was the dominant protein (Table 1). To further validate that the target recognized by G9 was SWP1, prokaryotic-expressed recombinant SWP1 (rSWP1) was purified (Fig. S3A), and the Western blotting results verified that the rSWP1 could be recognized by G9 mAb (Fig. 2B). Furthermore, rabbit polyclonal antibody was produced by utilizing rSWP1 as antigen and subsequently used in the Western blotting experiment (Fig. S3B). As expected, a 30-kDa protein band was recognized by rabbit polyclonal antibody in the above sample of immunoprecipitation, which was consistent with G9 mAb. The ELISA analysis was also performed, which implied the high specificity and affinity of the G9 mAb against SWP1 (Fig. S4). In summary, we demonstrated that the G9 mAb could efficiently bind to SWP1, which laid the foundation for the following neutralizing experiments.

**TABLE 1:** LC-MS/MS analysis of the protein of IP with mAb G9

Gene loci	Annotation	Cover percent	MM (kDa)	No. of unique peptides	No. of peptides	Unique peptidase
NBO_552g0005	SWP1	49.46%	32.09	9	29	K. ISAFTLIPVMDDR. R K. LFDHHEIEELAYELFIR. L K. YLCSSYGPNGYVSGDVFILR. N R. DVVLIVLNDFSHYLK. L R. IASVSAIM*ISSK. F R. LVASFLDSCSYPK. S R. NPNYEISTDYVSHEFNTTR. V R. SEDEELCTSPLAAFIK. L R. VVCDVNFPDLLCR. R
NBO_1522g0001	SWP8	9.82%	18.5	4	2	K. NVEIEVGK. G R. FSNDFQPK. D
NBO_7g0015	PTP2	5.4%	30.93	2	1	K. EAYVFNAIGEVLSTK. Q
NBO_52g0005	Hypothetical protein	27.94%	79.76	1	1	-. MDLSLRDIFYLLDMKNLPK. R
NBO_26g0013	SWP4	5.54%	40.73	1	1	K. LYNITVSIPSTTAGAAPTIR. N

### Cloning of G9 mAb gene and construction of scFvG9

**Fig 3:**
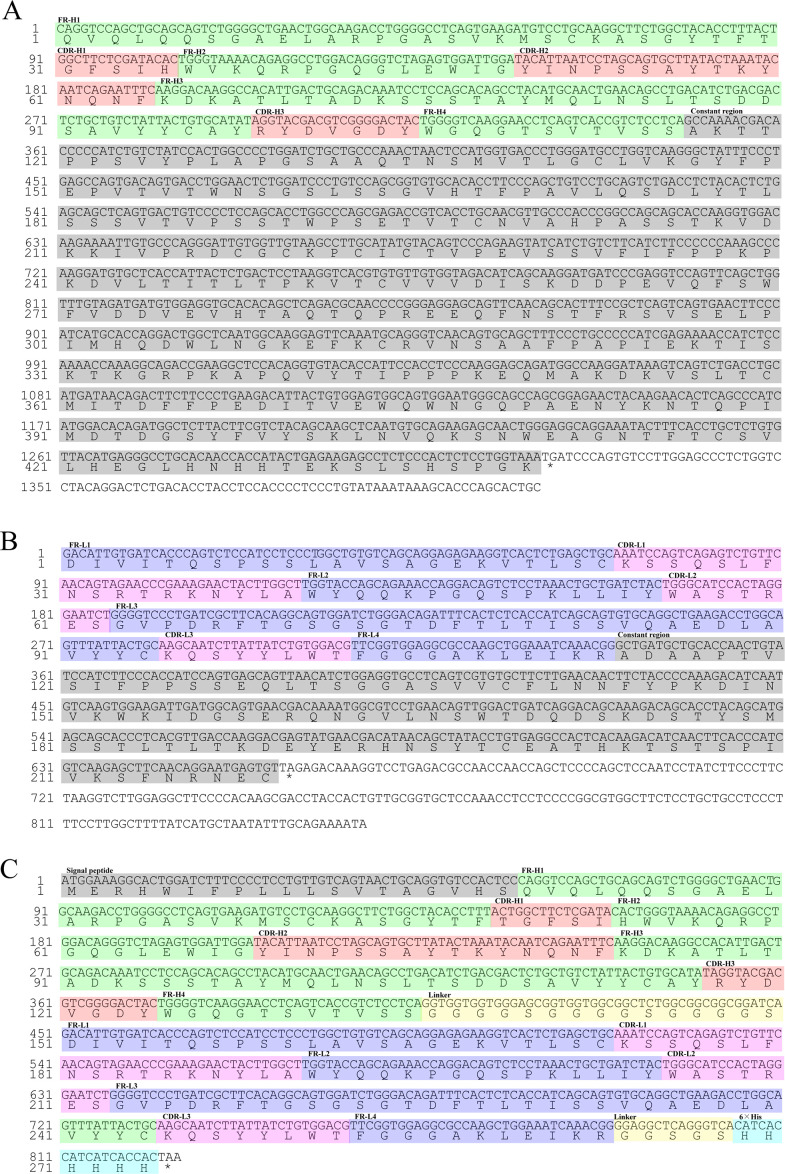
The clone of heavy chain and light chain of mAb G9 and the construction of scFvG9. Heavy-chain (**A**) and light-chain (**B**) regions were cloned from G9 hybridoma with degenerate primers. (A) The green is the framework regions (FRs) of heavy chain. Peach is CDRs of heavy chain. While the gray is constant region. (B) The lavender is the FRs of light chain. Pink is CDRs of light chain. While the gray is constant region. (C) scFVG9 was constructed using heavy-chain and light-chain variable region sequences which were joined by a glycine-serine (G4S)_3_ linker (yellow). A signal peptide (gray), CL40 anti-EBV/HHV-4 gHgL immunoglobulin heavy chain (MF104552.1), was cloned in the 5′-end, and a 6 × His tag (blue) was added into the 3′-end.

The mouse mAb subclass assay kit analysis showed that the G9 monoclonal antibody belongs to the IgG1 subclass (Fig. S2). Based on this, we designed degenerate primers and amplified G9 heavy-chain and light-chain gene sequences from cDNA that was reverse-transcribed from G9 hybridomas RNA. The sequencing results were analyzed using IgBLAST (https://www.ncbi.nlm.nih.gov/igblast/) and showed that the heavy chain and light chain of G9 mAb contained three classical complementarity-determining regions (CDRs) (Fig. 3A and B). Primers were designed based on the sequencing results, and overlap PCR was used to link the variable domains of G9 heavy chain and light chain by a (G4S)_3_ linker to obtain scFvG9 (Fig. 3C).

To detect the binding ability of scFvG9 to SWP1, the indirect ELISA was carried out to determine the titer of antibodies. The SWP1 gene was cloned into the pGEX-4T-1 vector to produce the fusion protein of GST-SWP1 (Fig. S5A), and the antibodies obtained using a baculovirus expression system were incubated with the expressed GST-SWP1 (Fig. S5B). Although the titer of the scFvG9 antibody was lower than that of the G9 intact antibody, it could specifically react with SWP1 protein (Fig. S5C).

### Expression of G9 and scFvG9 in BmE cells can inhibit *N. bombycis* proliferation

To evaluate the inhibitory effect of G9 on *N. bombycis*, silkworms were injected with mAb G9 and orally infected with *N. bombycis*. The results showed that *N. bombycis* load of the G9 antibody injected group was lower than that of negative serum-injected group in fat body, and the reduction in parasite loads was dose-dependent with the group receiving a higher dose (1 µg/larva) of the G9 mAb injection displaying a lower parasite load than the group receiving the mAb at a dose of 0.1 µg/larva. However, there was no evident effect on microsporidia load in midgut, which may be due to the inability of the injected antibody to penetrate into midgut effectively (Fig. S6).

**Fig 4:**
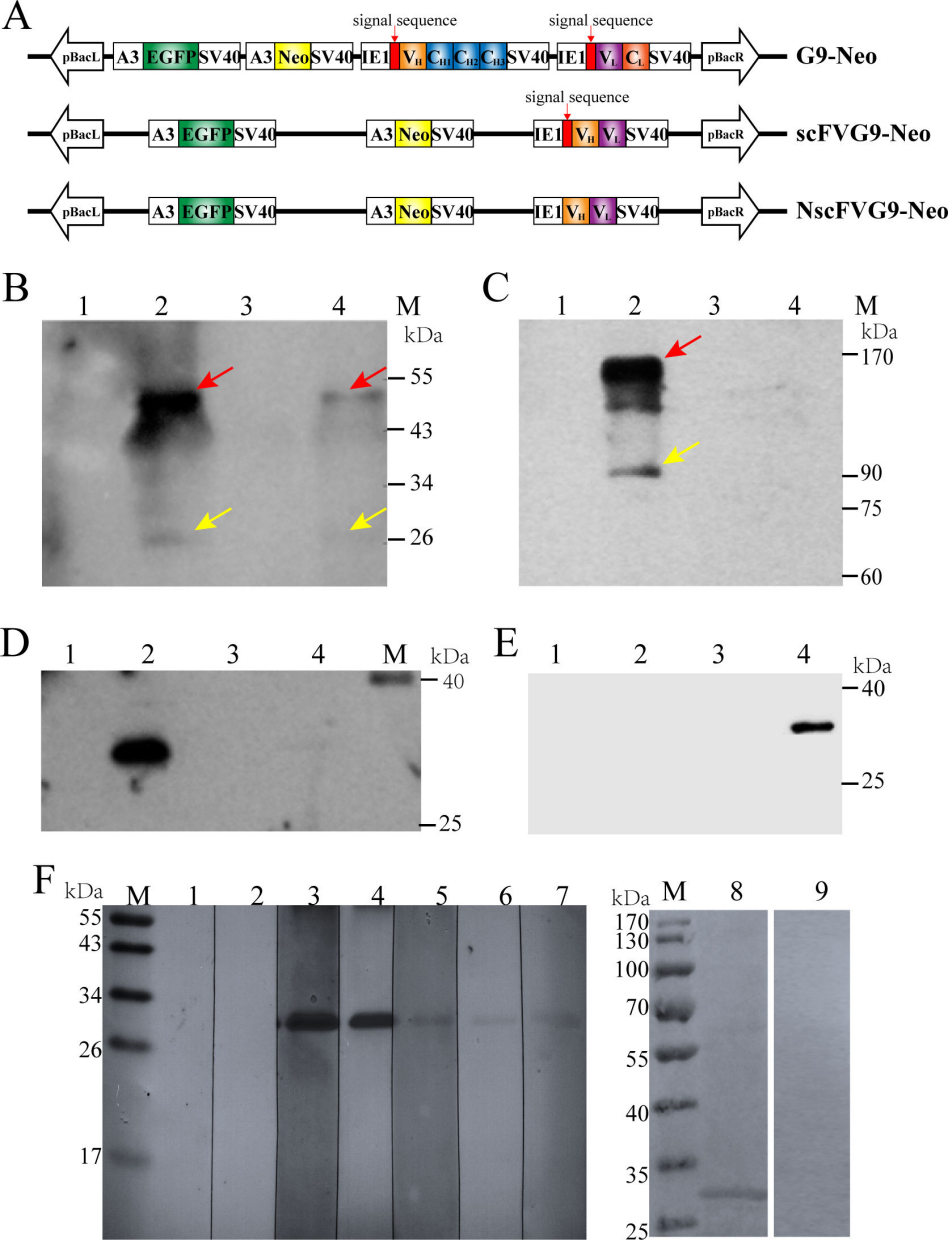
Analysis of antibodies expression and their specificity. (A) Schematic diagram of vectors construction for establishing transgenic cell lines. (B) Detection of G9 expression by SDS-PAGE. There are heavy-chain (the red arrow) and light-chain (the yellow arrow) hybridization signal bands in G9-Neo cells culture supernatant (line2) and G9-Neo cells lysate protein (line4) while not in BmE cells culture supernatant (line1) and cells lysate (line3). (C) Detection of G9 expression by Native-PAGE. The heterodimer (the yellow arrow) and tetrameric (the red arrow) hybridization signal bands of heavy and light chains were observed in G9-Neo cells culture supernatant (line2), while it is failed in G9-Neo cells lysate (line4). There are also no signal in BmE cells culture supernatant (line1) and cells lysate (line3). (D) Detection of scFvG9 expression by SDS-PAGE. The signal of scFvG9 was observed in scFvG9-Neo cells culture supernatant (line2), while it is failed in scFvG9-Neo cells lysate (line4). There are also no signal in BmE cells culture supernatant (line1) and cells lysate (line3). (E) Detection of NscFvG9 expression by SDS-PAGE. The signal of scFvG9 was observed in NscFvG9-Neo cell lysate (line4), while it is failed in NscFvG9-Neo cells culture supernatant (line2). There are also no signal in BmE cells culture supernatant (line1) and cells lysate (line3). (F) The specificity of G9, scFvG9, and NscFvG9. Spore proteins were separated by SDS-PAGE and then transferred to PVDF membranes. The cell culture supernatant of G9-Neo (line 4), cell lysate of G9-Neo (line 5), cell culture supernatant of scFVG9-Neo (line 6), cell lysate of scFVG9-Neo (line 7), cell lysate of NscFVG9-Neo (line 8), and cell culture supernatant of scFVG9-Neo (line 9) were used as the primary antibody. While cell lysate (line 1) and cell culture supernatant (line 2) of BmE cells were used as negative controls, mAb G9 (line 3) was used as positive control. M, protein marker.

Next, the transgenic cells expressing antibodies were constructed to assess the inhibitory effect of microsporidia. IE1 promoter was used to drive the expression of the G9 mAb heavy-chain (G9H) and light-chain (G9L) genes in the G9-Neo vector (Fig. 4A). As scFv only includes the antigen-binding site, its molecular weight is 1/6 of the intact antibody molecule; therefore, scFv has greater permeability due to its lower molecular weight compared to the intact antibody. Since microsporidia could be present intracellularly or extracellularly during silkworm infection, we constructed the expression vectors with or without the secretion signal peptide (CL40 anti-EBV/HHV-4 gHgL immunoglobulin heavy Chain, MF104552.1), named scFvG9-Neo and NscFvG9-Neo, respectively (Fig. 4A). G418 was used to screen and obtain G9-Neo (intact antibody secreted into the extracellular matrix), scFvG9-Neo (nanoantibody secreted into the extracellular matrix), and NscFvG9-Neo (nanoantibody expressed intracellularly) transgenic cell lines (Fig. S6 and S7). Western blotting results demonstrated that G9, scFvG9, and NscFvG9 were successfully expressed in BmE cells (Fig. 4). G9H and G9L monomer hybridization signals were detected in denaturing protein gel (Fig. 4B), and a hybridization signal was observed at approximately 170 kDa in a non-denaturing gel, which was tetramers of G9H and G9L (Fig. 4C), suggesting that G9H and G9L were expressed correctly in silkworm cells and could bind and form a functional tetrameric structure. A specific band of scFvG9 was detected in scFvG9-Neo cells culture supernatant (Fig. 4D), while it was detected in NscFvG9-Neo cells lysate (Fig. 4E).

To validate the activity and specificity of the expressed antibody, the alkali-soluble germination proteins were subjected to SDS-PAGE and the expressed antibodies (G9, scFvG9, and NscFvG9) were used as primary antibody. The result showed that all three species of the expressed antibodies could recognize SWP1, and G9 and scFvG9 antibodies were mainly present in the culture medium, while NscFvG9 was present intracellularly, which was consistent with the expected results (Fig. 4F).

**Fig 5:**
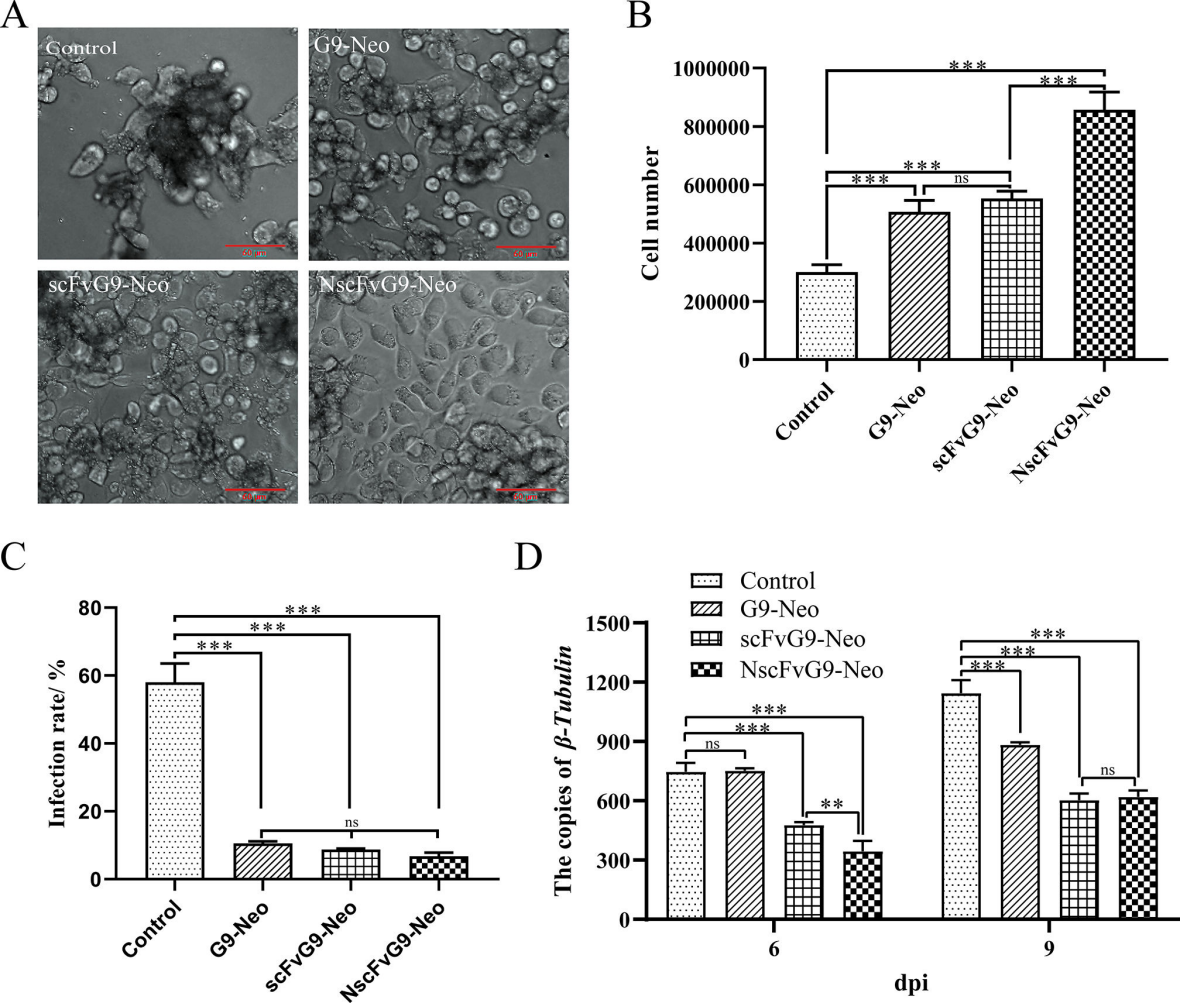
Inhibition effect of *N. bombycis* on G9-Neo, scFvG9-Neo, and NscFvG9-Neo cell lines. (A) The morphology of BmE cells and transgenic cells at 9 days after *N. bombycis* infection. The cell status of G9-Neo, scFvG9-Neo, and NscFvG9-Neo was much better than that of BmE cells. (B) The number of all transgenic cells exceeded that of the control, while the cell number of NsFvG9-Neo was more than that of G9-Neo and scFvG9-Neo. (C) The infection rate of transgenic cells was lower than that of the control, while there was no significant difference between transgenic cells. (D) The proliferation of *N. bombycis* was measured by qPCR. The total genomic DNA was extracted from the cells infected with microsporidia at 6 or 9 dpi. The copies of *β-tubulin* indicated the number of *N. bombycis*. Vertical bars represent the mean ± SEM (*n* = 3). **P < 0.01, ***P < 0.001.

To analyze the anti-microsporidia activity of the expressed antibodies, *N. bombycis* was added to three transgenic cell lines for infection and normal BmE cells were used as a control. By day 9 post infection, the growth states of transgenic cells were better than that of the control (Fig. 5A). Additionally, the total cell counts of three transgenic cell lines were significantly higher than that of the control BmE cells and the count of NscFvG9 expressing cell line was higher than those of G9-Neo and scFvG9 cell lines (Fig. 5B). Meanwhile, DAPI staining was used to calculate the pathogen infection rate (Fig. S6), and the statistical result was shown in Fig. 5C. As shown, the infection rate of G9-Neo, scFvG9-Neo, and NscFvG9-Neo cell lines was approximately 10%, while the infection rate of BmE cell line was about 60%, which suggested that the transgenic cells were able to significantly inhibit *N. bombycis* proliferation (Fig. 5C).

The copies of *N. bombycis β-tubulin* were also used to detect the pathogenic load by qPCR. The results showed that the pathogenic load in the cell lines expressing scFv was significantly lower than that of the control group at day 6 and 9 post infection, with the NscFvG9-Neo cells exhibiting higher reduction than that of the scFvG9-Neo cells. Moreover, the pathogenic load of the G9-Neo cell line was significantly lower than that of the control group only at 9 dpi but not at 6 dpi (Fig. 5D). These findings suggested that the expression of the G9 intact antibody and scFvs in cells could reduce *N. bombycis* infection, and NscFvG9 had a strong inhibitory effect than other antibodies.

### Resistance of the transgenic silkworm to *N. bombycis*

**Fig 6:**
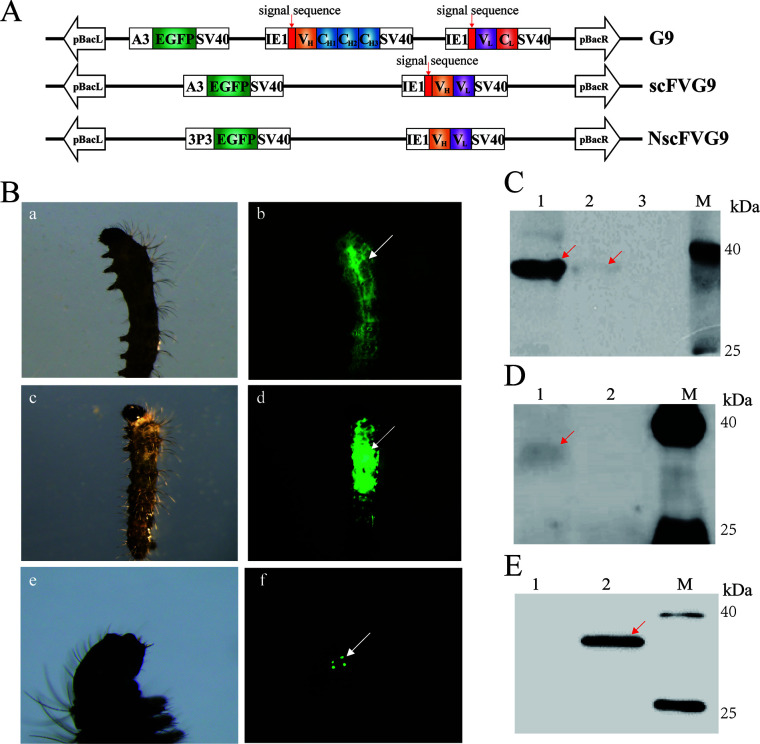
Verification of transgenic silkworm. (A) Schematic diagram of vectors construction for establishing transgenic cell lines. (B) Fluorescent screening of G9 (**A, B**), scFvG9 (**C, D**), and NscFvG9 (**E, F**) transgenic silkworm. Arrows indicated the signals of EGFP protein. (C–E) The rSWP1 was analyzed by SDS-PAGE for verification of G9, scFv9, and NscFvG9 specificity. G9 (**C**), scFv9 (**D**), and NscFv9 (**E**) transgenic silkworms were used as the primary antibody. (C) A strong hybridization signal was detected in the hemolymph (line 1) and a weak hybridization signal in the cell lysate (line 2) of G9 transgenic silkworm, while no hybridization signal in the hemolymph of control silkworm (line 3). (D) There was a hybridization signal in the hemolymph of scFv9 transgenic silkworm (line 1), while no hybridization signal in the cell lysate of scFv9 transgenic silkworm (line 2). (E) In NscFv9 transgenic silkworm, the cell lysate (line 2) had a hybridization band but not the hemolymph (line 1).

To further evaluate the inhibitory effects of different forms of G9 antibodies against *N. bombycis*, we constructed three types of transgenic silkworms. These silkworms express intact antibody (G9), scFv in secreted form (scFvG9), or scFv antibody in intracellulary form (NscFvG9), respectively (Fig. 6A and B). The expressions of antibodies in silkworm were confirmed by Western blotting. The rSWP1 was separated by SDS-PAGE and the hemolymph or cell lysate of silkworm, which were used as primary antibody. In G9 transgenic silkworm, a strong hybridization signal was detected in the hemolymph and a weaker hybridization signal in the cell lysate (Fig. 6C). In scFv9 transgenic silkworm, a hybridization signal was detected in the hemolymph but not in cell lysate (Fig. 6D). While in NscFv9 transgenic silkworm, a hybridization band was detected in the cell lysate but not in hemolymph (Fig. 6E). In summary, our results showed that the G9, scFvG9, and NscFvG9 transgenic silkworms could express the antibodies that recognized the microsporidia spore wall protein SWP1.

**Fig 7:**
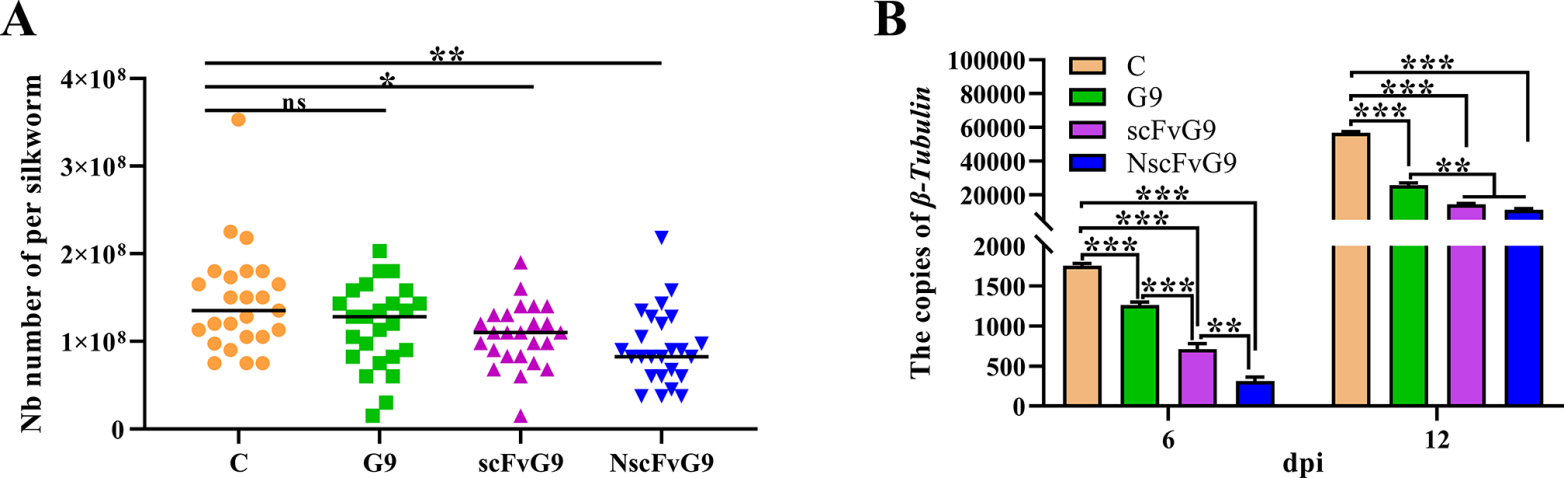
Inhibition effect against *N. bombycis* in G9, scFv9, and NscFv9 transgenic silkworm. (A) After *N. bombycis* infection at 12 days, the number of spores per silkworm was counted. The number of each group was 25. *P < 0.05, **P < 0.01. (B) The proliferation of *N. bombycis* was measured by qPCR. The total genomic DNA was extracted from the silkworms at 6 or 12 dpi. The *N. bombycis β-tubulin* gene was used to detect pathogen load. Vertical bars represent the mean ± SEM (*n* = 3). *P < 0.05, ***P < 0.001.

Next, we evaluated the inhibitory effect of transgenic silkworm against *N. bombycis* infection. The *N. bombycis* spores was fed to these three transgenic silkworm lines, while the wild silkworms were used as a control. Pathogen proliferation was evaluated by counting the number of pathogens per silkworm. The result showed that the microsporidia load was significantly decreased in scFvG9 and NscFvG9 silkworms compared with the control group and the NscFvG9 expressing silkworms exhibited a stronger reduction than the scFvG9 expressing silkworms. Regrettably, there was no significant difference between the silkworm expressing G9 intact antibody and the control silkworm (Fig. 7A). We found that, in general, the pathogenic loads were substantially decreased in transgenic silkworms compared with the control group at 6 and 9 dpi, with NscFvG9-expressing silkworms harboring the lowest microsporidia load following by the scFvG9-expressing silkworms at 6 dpi (Fig. 7B). Our result with the inhibitory effect of *N. bombycis* in transgenic silkworm was largely consistent with what we observed in the transgenic cells (Fig. 4), suggesting that the scFv without the signal peptide was more effective than the other antibodies in inhibiting the proliferation of *N. bombycis* at both cellular and individual host level.

## DISCUSSION

The first microsporidia species to be named was *N. bombycis* from *B. mori*, in 1857, and a great number of other species have subsequently been identified. For example, *N. ceranae* causes honeybee colony collapse (4, 5), and *Enterocytozoon hepatopenaei* frequently causes substantial economic losses to the shrimp-farming industry (32, 33). Some microsporidia in mammals are zoonotic pathogens, which threaten the human health (16, 34). Despite tremendous advancements in our understanding of the gene functions of microsporidia and the interactions between hosts and pathogens, there is still a lack of effective treatments for treating microsporidiosis. In the study of *P. falciparum* infection, the expression in mosquitoes of monoclonal antibody or scFvs targeting the surface proteins of *P. falciparum* could significantly decrease the infection levels (22, 27, 30, 35). However, monoclonal antibody or scFv antibody has rarely been utilized in treating microsporidia infection. In our study, we demonstrated that the monoclonal antibody and targeting *N. bombycis* spore wall proteins could be successfully developed by using germination fluid as an immunization antigen and subsequently utilized as a therapeutic agent for *N. bombycis* infection in silkworms. We postulate that at least two different mechanisms might be involved in the inhibitory effects mediated by the antibodies targeting *N. bombycis* spore wall proteins. First, the spore wall protein-specific antibody may block the formation of microsporidia spore wall, resulting in reduced parasite attachment and infection (36). Second, the adhesion of microsporidia to host cells may be blocked by the presence of spore wall-specific antibodies (37).

The scFv is formed by linking the heavy-chain variable domain and light-chain variable domain with a 15 amino acid linker peptide. Such antibodies can retain affinity and activity toward the antigen and have low molecular weight, high permeability, and weak antigenicity. Our previous study on the scFvG4 showed potent inhibitory effects against microsporidia in the cellular level (38, 39). Recently, the scFv antibodies against the outer loops of *N. bombycis* ATP/ADP-transporters could efficiently inhibit the parasite growth in Sf9 cell line (40). In the current study, we used the spore germination fluid proteins of *N. bombycis* as immunogens to prepare monoclonal antibody. The monoclonal antibody G9 was developed, and the expression of this antibody in silkworms significantly inhibited *N. bombycis* proliferation. Our present study demonstrated the scFv without the secretory signal peptide exhibited more potent inhibitory effects than the scFv with a signal peptide. This may be because extracellular mature spores are less effectively suppressed by the antibodies targeting spore wall proteins. In contrast, when microsporidia reside intracellularly in the hosts, the expression of spore wall targeting antibody in the host may effectively prevent microsporidia from maturing in host cells.

It may still be necessary to improve the inhibitory effects of the developed antibodies expressed in target hosts, especially in the transgenic silkworm expressing the G9 intact antibody. Even though the highly expressed constitutive promoter was used to drive the expression of the G9 antibody in silkworms, the resulting expression level was still relatively low, which may limit its function. Furthermore, the binding affinity of the G9 antibody to SWP1 may need to be further improved to enhance its, resulting in that this expression level of antibody was also insufficient to neutralize the pathogen neutralizing activity. Lastly, the target protein, SWP1, was identified to be localized on the spore surface and has been shown to bind to deproteinated chitin spore coat (41). Although SWP1 of *Encephalitozoon cuniculi* (EnP1; *E. cuniculi* ECU01_0820) may play functions both in a structural capacity and in adhesion to and infection host cells (42), further research is still necessary to determine that SWP1 of *N. bombycis* was directly associated with microsporidia proliferation. The infection of microsporidian begins with the ejection of the polar tube and releasing of sporoplasm, which subsequently infected the host cells (43). During this period, microsporidia lack spore wall protection. Therefore, it may be the most susceptible stage for intervention. To prepare antibodies against the surface protein of microsporidia sporoplasm might be a better target. In addition, additional factors, such as instability, short half-life, and easy degradation in silkworm, also potentially reduce the inhibitory effect of antibodies on microsporidia. More importantly, our study showed that the pathogenic loads were substantially decreased in antibody-expressing silkworms, especially in NscFvG9-expressing silkworms, which provided the essential groundwork for the future development of *N. bombycis-*resistant transgenic silkworms and novel strategies for other microsporidia control.

### Conclusion

In conclusion, there are a few reports on the therapies for infections caused by *N. bombycis* and other microsporidia (44, 45). We used germination fluid as immunogens to prepare a monoclonal antibody, and the corresponding scFvs exhibited a capacity for blocking microsporidia infection. Expression of high level of scFvs targeting *N. bombycis* in silkworms upon microsporidia infection may be valuable in agricultural production. Additionally, we constructed antibody-expressing transgenic insects for controlling *N. bombycis* infection and established a useful methodology with potential applications for treating other microsporidia diseases. Lastly, we demonstrated that the germination fluid prepared by exposing microsporidia to alkaline buffer can be utilized as an immunogen for the development of antibodies with therapeutic applications, and similar strategies might be employed to other microsporidia.

## MATERIALS AND METHODS

### Insect rearing and cell lines

The *Bombyx mori* strain 871 was reared on fresh mulberry leaves and maintained at 25°C under a photoperiod of 12 h light and 12 h dark.

BmE, a *B. mori* cell line, was cultured in Grace culture (Gibco, USA) with 10% fetal bovine serum and 1% penicillin/streptomycin (Gibco, USA) and maintained at 28°C.

The Sf-900 III cell was cultured in Sf-900 III SFM (Gibco, USA) and maintained at 28°C.

### *N. bombycis* extraction and purification

A total of 10^8^ spores/mL of *N. bombycis* spores (CVCC 102059) were evenly applied to mulberry leaves and allowed to dry before the leaves were fed to the third instar *Bombyx mori* larvae (1,000 spores/larvae). Silk glands were collected in the late fifth instar and homogenized using a homogenizer. Physiological saline (0.85%) was added to the homogenate, and the homogenate was filtered through gauze and collected. The filtrate was centrifuged to obtain a crude extract, which was subsequently subjected to Percoll (Cytiva, Sweden) density gradient centrifugation at 13,000 *g* for 30 min for fine purification. The bottom pellet of purified spores was collected.

### Preparation of *N. bombycis* alkali-soluble germination protein

A total of 5 × 10^8^ spores were added to 50 µL 0.1 mol/L K_2_CO_3_ and evenly mixed. The solution was incubated in a 28°C water bath for 60 min and evenly mixed by shaking every 10 min. The solution was centrifuged at 10,000 *g* for 5 min, and the supernatant was alkali-soluble germination proteins without spore coat from the potassium carbonate treatment.

The rate of spore germination was detected by Giemsa staining. Spore samples before and after germination treatment were applied on a clean glass slide and allowed to dry at room temperature. Then, the samples were fixed using Carnoy’s fixative for 10 min, followed by staining with Giemsa for 30 min.

### Preparation of monoclonal antibodies

All animal experiments were conducted according to the guidelines of the experimental animal ethics review committee of Southwest University, which have approved this study (Permit Number: AERCSWU2017-7). Female BALB/c mice (6–8 weeks old) were used and 100 µg alkali-soluble germination protein (*N. bombycis* was treated with K_2_CO_3_ and the spore walls were removed by centrifugation), and Freund’s complete adjuvant or Freund’s incomplete adjuvant were mixed in a 1:1 ratio and injected subcutaneously four times at multiple spots on the mice. Monoclonal antibodies were developed based on the previously described method (46). Based on manufacturer recommendations, a mouse monoclonal antibody isotype assay kit (Roche, Switzerland) was used to identify the antibody subclass.

### Immunofluorescence assay

Paraformaldehyde (4%) was used to fix purified *N. bombycis* on a glass slide followed by permeabilization for 10 min at room temperature using 0.5% Triton X-100. A PBS-bovine serum albumin was used for blocking for 60 min. Then, the slides were incubated with mAb diluted with PBS-bovine serum albumin at 37°C for 60 min (dilution: 1:500) and washed with PBS, while negative serum was used as a control. Finally, secondary antibody labeled with Alexa488 and DAPI were diluted with IFA blocking solution (1:1,000) and incubated at dark for 40 min. After washing, the slides were sealed and observed under confocal laser scanning microscope (Olympus, Japan).

### Enzyme-linked immunosorbent assay

For the ELISA test, plates were coated with alkali-soluble protein isolated from *N. bombycis* (0.5 µg/well) or recombinant SWP1 protein (0.3 µg/well) for overnight at 4°C and then washed 5 min with PBST buffer for three times. The plates were blocked for 2 h with 5% skimmed milk, and then the mAb was added and incubated for 1 h at room temperature, while the negative serum was used as a control. After washing three times with PBST buffer, peroxidase-labeled goat anti-mouse IgG (Sigma, USA) was added to the plates and incubated for 1 h at room temperature. Then, TMB Horseradish Peroxidase Color Development Solution (Beyotime, China) for ELISA was added. The reaction was stopped by adding 2 M H_2_SO_4_ and optical density at 450 nm was measured for each well with a microplate reader (TECAN, Switzerland). For the indirect ELISA test, plates were coated with recombinant GST-SWP1 protein (0.3 µg/well) for overnight at 4°C and then washed with PBST buffer. Five percent of skimmed milk was used to block for 2 h. Then, the recombinant antibodies fused 6x-His tag were added and incubated for 1 h at room temperature, while the negative serum was used as a control. After washing three times with PBST buffer, 6x-His tag rabbit monoclonal antibody (ThermoFisher, USA) was added to the plates and incubated for 1 h at room temperature. After washing with PBST buffer, peroxidase-labeled goat anti-rabbit IgG (Sigma, USA) was added to the plates and incubated for 1 h at room temperature. Then, TMB Horseradish Peroxidase Color Development Solution (Beyotime, China) was added and 2 M H_2_SO_4_ was used to stop the reaction. OD_450nm_ was measured for each well with a microplate reader (TECAN, Switzerland).

### Immunoprecipitation and liquid chromatography-tandem mass spectrometry analysis

The *N. bombycis* alkali-soluble proteins were buffered to a pH of about 7 with hydrochloric acid, and the total protein was quantitated using Bradford method. Then, *N. bombycis* alkali-soluble proteins (4 µg) were incubated with the monoclonal antibody (4 µL) at 4°C overnight, while the serum of non-immune mouse was used as a control. Subsequently, the alkali-soluble proteins-antibody complexes were incubated with 40 µL Protein A/G plus Agarose (Beyotime Biotechnology, China) at 4°C overnight. The beads were collected by centrifugation and then washed with PBS buffer for five times. The proteins were incubated with 100 µL 5 × SDS loading buffer (Beyotime Biotechnology, China) at 100°C for 10  min. The supernatants were collected for SDS-PAGE analysis.

After SDS-PAGE separation, the gel was stained with silver nitrate, and the different band was cut and subjected to protein identification using LC-MS/MS as described previously (36).

### Western blotting analysis

For the SDS-PAGE, protein samples were mixed with SDS-PAGE protein loading buffer (Beyotime, China), heated at 100°C for 10 min, and fractionated on an SDS-PAGE gel. For the Native-PAGE, protein samples were mixed with native gel sample loading buffer (Beyotime, China) and fractionated on the Native-PAGE gel. After that, the proteins were transferred onto PVDF membranes (Roche, Switzerland). Subsequently, 5% defatted milk in TBST (150 mM NaCl, 20 mM Tris-HCl, 0.05% Tween-20) was employed to block the membranes. Then, it was incubated with primary antibody, mAb G9, or negative serum for 1 h. After washing three times with TBST, HRP-labeled goat anti-mouse IgG (Bio-Rad, USA) was added and incubated for 1 h. Sequentially, ECL Plus Western Blotting Detection Reagents (Bio-Rad, USA) were employed to detect the bound antibodies.

### Expression and purification of recombinant SWP1 protein of *N. bombycis* and preparation of rabbit polyclonal antibody

The gene of SWP1 was cloned by PCR from cDNA of *N. bombycis* and inserted into pET30a vector following *Bam*H I and *Hin*dIII restriction enzyme digestion. Then, the vector for expression of the recombinant SWP1 (rSWP1) was transformed into *E. coli* Rosetta. When the culture reached an OD600 of 0.4–0.6, the recombinant bacteria was induced with 0.1 mM isopropyl-β-d-thiogalactopyranoside for 20 h. The cells that contained recombinant vector were resuspended in lysis buffer (20 mM Tris-HCl, pH 8.0, and 100 mM NaCl) and sonicated. rSWP1 protein was purified using the Ni–NTA beads according to the manufacturer’s instructions (QIAGEN, USA).

Then, the 12-week-old New Zealand white rabbit was immunized with purified rSWP1 to generate polyclonal antibodies. rSWP1 protein (400 µg) in Freund’s complete adjuvant (Sigma, USA) was injected for the first immunization. Then, rSWP1 protein in Freund’s incomplete adjuvant (Sigma, USA) was used as the immunogen for the second and third immunization with respective intervals. The rabbit was subcutaneously injected every 14 days, and 7 days after the last injection, serum was collected from rabbit blood and purified.

### mAb G9 gene cloning

The total RNA was isolated using the total RNA extraction kit (OMEGA, USA) from G9 hybridoma cells and the contaminating genomic DNA was digested with RNase-free DNase I (Takara, Japan). Then, total RNA was used to reverse transcribe the first-strand cDNA using Oligo dT Primer with M-MLV Reverse Transcriptase (Promega, USA). The degenerate primers H-F (47) and 3′ Racer Primer were used for amplification of the heavy chain gene of G9 monoclonal antibody, while primers L-F (48) and 3′ Racer Primer were used for amplification of light chain gene. Q5 high-fidelity polymerase was used for PCR amplification. The PCR conditions used were pre-denaturation at 98°C for 30 s; followed by 30 cycles of denaturation at 98°C for 15 s, annealing at 55°C for 30 s, and extension at 72°C for 2 min); followed by final extension at 10 min. Finally, the G9 heavy chain (G9H) and light chain (G9L) genes were ligated to pMT19-T for sequence analysis. All primers were listed in Table S2.

### Vector construction

The analysis results of G9 mAb gene sequences were used to design different primers (Table S2) to amplify the G9 heavy chain, light chain, and scFv sequences. The primers G9H-F and G9H-R were used to amplify the heavy chain of G9 (G9H) containing a signal peptide in the 5′ -end and a 6 × His tag in the 3′-end. Similarly, primers G9L-F and G9L-R were used to amplify the light chain of G9 (G9L). The scFvG9H-F and scFvG9H-R primers were used to obtain the variable domain of heavy chain (VH) containing a signal peptide. scFvG9L-F and scFvG9L-R primers were used to obtain the variable domain of light chain (VL). Overlap PCR was used to link VL and VH through a (G4S)_3_ linker and obtain scFvG9. The signal peptide of VH was removed to obtain NscFvG9. All PCR products were ligated to pMD19-T for sequencing to obtain positive clones. G9H, G9L, scFvG9, and NscFvG9 were digested with *Bam*H I and *Not* I before ligation to pSL[IE1-MCS-SV40] vector to obtain pSL[IE1-G9H-SV40], pSL[IE1-G9L-SV40], pSL[IE1-scFvG9-SV40], and pSL[IE1-NscFvG9-SV40], respectively. The pSL[IE1-G9L-SV40] was digested with *Eco*RI and *Hin*dIII to obtain the IE1-G9L-SV40 expression cassette, which was inserted into pSL[IE1-G9H-SV40] to obtain pSL[IE1-G9H-SV40 + IE1-G9L-SV40]. The above constructed expression cassettes were digested with *Asc*I and inserted into pBac[A3-EGFP + A3-Neo] to obtain pBac[A3-EGFP + A3-Neo + IE1-G9H + IE1 G9L] (G9-Neo), pBac[A3-EGFP + A3-Neo + IE1-scFvG9] (scFvG9-Neo), and pBac[A3-EGFP + A3-Neo + IE1-NscFvG9] (NscFvG9-Neo) for construction of stable expression cell lines. For the construction of transgenic silkworms, [IE1-G9H-SV40 + IE1-G9L-SV40] and IE1-scFvG9-SV40 expression cassettes were inserted into pBac[A3-EGFP] obtain pBac[A3-EGFP + IE1-G9H + IE1 G9L] (G9) and pBac[A3-EGFP + IE1-scFvG9] (scFvG9), while the IE1-NscFvG9-SV40 expression cassette was inserted into pBac[3 × P3-EGFP] to obtain pBac [3 × P3-EGFP + IE1-NscFvG9] (NscFvG9).

### Expression and purification of recombinant GST-SWP1 protein and recombinant antibodies

The gene of SWP1 was cloned by PCR and inserted into pGEX-4T-1 vector following *Bam*H I and *Xho*I restriction enzyme digestion. Then, the vector for expression of the recombinant GST-SWP1 was transformed into *E. coli* Rosetta. When the culture reached an OD600 of 0.4–0.6, the recombinant bacteria was induced with 0.1 mM isopropyl-β-d-thiogalactopyranoside for 20 h. The cells that contained recombinant vector were resuspended in PBS buffer and sonicated. rGST-SWP1 protein was purified using the Glutathione Sepharose High Performance according to the manufacturer’s instructions (GE HealthCare, USA).

The recombinant antibodies were produced by Bac-to-Bac Baculovirus Expression System. The heavy chain of G9 (rG9H), containing a signal peptide in the 5′ -end and a 6 × His tag in the 3′-end, was cloned into pFastBac Dual vector following *Bam*H I and *Not* I and driven by polyhedrin promoter. Then, light chain of G9 (rG9L) was driven by p10 promoter following *Xma* I and *Kpn* I restriction enzyme digestion. The expression cassettes of rG9H and rG9L formed pFast-rG9 vector. scFvG9, containing a signal peptide and a 6 × His tag, was also cloned into pFastBac Dual vector following *Bam*H I and *Not* I and driven by polyhedrin promoter. The vector of pFast-rG9 or pFast-rscFvG9 were transformed into DH10Bac *E. coli* cells and the recombinant bacmid DNA was extracted. Then, the recombinant bacmid DNA was transfected into Sf-900 III cell to get P0 recombinant baculovirus. P0 recombinant baculovirus-infected insect cells to amplify virus and the high titer of P2 virus obtained from transfecting Sf9 cells using P1 virus. The P2 virus was used to infect insect cells for expression of the recombinant protein and serum-free medium was collected. The recombinant antibodies were purified using the Ni–NTA beads according to the manufacturer’s instructions (QIAGEN, USA).

### Establishment of antibody-expressing cell lines and immune challenge

BmE cells were transfected with the expressing antibodies plasmid (G9-Neo, scFvG9-Neo, or NscFvG9-Neo) and the helper plasmid pHA3PIG using Cellfectin II DNA Transfection Reagent (Gbico, USA). Three days later, the cells were cultured in Grace medium containing 800 µg/mL Geneticin (G-418; Merck, Germany), and the culture medium was changed every 2 days. The screening process lasted for about 4 months.

Then, the transgenic cells and the control BmE cells were infected by purified *N. bombycis* mature spores (spores: cells ratio 10:1). Six and nine days after infection, different cell lines were collected and used to extract genomic DNA (OMEGA, USA) for qPCR. Nine days after infection, different cell lines were observed and cell numbers were determined by a haemacytometer under microscope (Olympus, Japan). All samples were run in triplicate.

### Generation of germline transgenic silkworms and immune challenge

Silkworm eggs for microinjection were collected within 2–6 h after they were oviposited. The vector (G9, scFvG9, or NscFvG9) and helper plasmid pHA3PIG were mixed in a 1:1 ratio and injected into embryos. After injection, the eggs were incubated in a 25°C and 75% relative humidity thermostatic incubator. The hatched larvae were reared with fresh mulberry leaves at 25°C under standard conditions. After larvae developed into adult moths, they were crossed to wild-type moths. After spawning, the G1 progeny were scored for the presence of positive transgenic individuals by different fluorescent markers using fluoresce microscopy (Olympus, Japan).

The fourth instar positive transgenic silkworms were orally infected with *N. bombycis* spores (10^4^/larva), while wild silkworm strain was used as a control. Twenty-five silkworms of each strain were individually homogenized by homogenizer 12 days after oral challenge. Then, the number of *N. bombycis* spores in each silkworm was counted with hemocytometer measurement. Six and 12 days after infection, different silkworms were collected and used to extract genomic DNA (OMEGA, USA) for qPCR. All samples were run in triplicate.

### Real-time quantitative PCR analysis

The standard curve of *β-tubulin* was established to count *N. bombycis*. The standard template used was described in previous research (46) and standard curve covered six orders of magnitude (1 × 10^2^–10^7^). The 20 µL mixture included 2 µL standard template or genomic DNA (50 ng/µL), 0.5 µL of each primer (10 mM; Table S2), 10 µL SYBR Green Master Mix reagent (Yeasen, Shanghai, China), and 7 L ddH2O. qPCR was performed according to the following parameters: one cycle of an initial denaturation step at 95°C for 5 min, followed by 40 cycles at 95°C for 10 s, 60°C for 20 s, and 72°C for 40 s. These experiments were repeated three times, and all samples were run in triplicate of each time.

### Statistical analyses

Statistical analyses were performed using GraphPad Prism 8 software (GraphPad, USA), and the significance of difference was determined by one-way ANOVA followed by a Tukey’s multiple comparison test.

## SUPPLEMENTAL MATERIAL

Supplemental material spectrum.00681-23-s0001.pdf Fig. S1 to S9; Tables S1 and S2.

ASM does not own the copyrights to Supplemental Material that may be linked to, or accessed through, an article. The authors have granted ASM a non-exclusive, world-wide license to publish the Supplemental Material files. Please contact the corresponding author directly for reuse.
