# Supplementary material for: A monoclonal antibody targeting spore wall protein 1 inhibits the proliferation of Nosema bombycis in Bombyx mori
Source: Microbiol Spectr. 2023 Oct 9;11(6):e00681-23. doi: 10.1128/spectrum.00681-23 (PMC10714992; doi:10.1128/spectrum.00681-23)
Supplement: Supplemental material — Fig. S1 to S9; Tables S1 and S2. [file spectrum.00681-23-s0001.pdf]

Supplemental Material

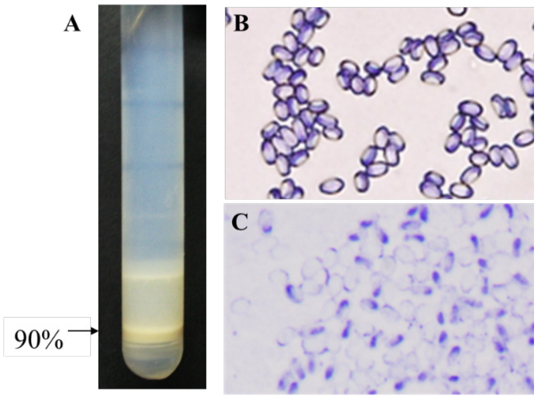

Fig. S1. Purification and germination of *N. bombycis*. A: Purification of spores by density gradient centrifugation. B: The Giemsa-staining of *N. bombycis* before treatment. C: The Giemsa-staining of *N. bombycis* treated by 0.1 mol/L K_2CO_3.

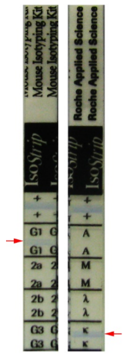

Fig. S2. Subtypes identification of mAb G9. The left red arrow indicated the subtype of heavy-chain was IgG1, while the right arrow showed the light-chain subtype was kappa (κ). The blue line in the '+' region is control line.

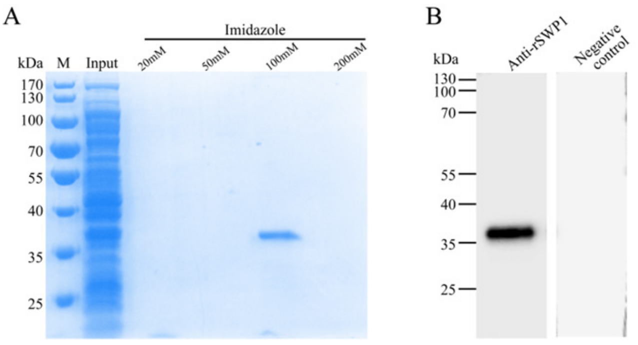

Fig. S3. Purification of rSWP1 and identification of SWP1 polyclonal antibody. Recombinant SWP1 was eluted by elution buffer contained different concentration imidazole. The

SDS-PAGE showed rSWP1 was purified from recombinant *E. coli* Rosetta by affinity chromatography, and the most of rSWP1 was eluted in elution buffer which contained 100mM imidazole (A). The polyclonal antibodies were obtained by immunized of rabbit with purified rSWP1, and the rabbit polyclonal antibodies could specifically recognize rSWP1 (B).

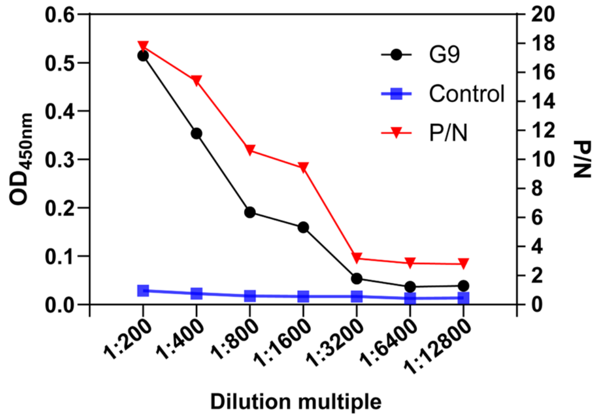

Fig. S4. Measurement of G9 titer. The mAb G9 titer was detected by ELISA, while negative serum was used as negative control. The ELISA plate was coated with rSWP1. A positive/negative (P/N) value higher than 2.0 was determined as positive standard.

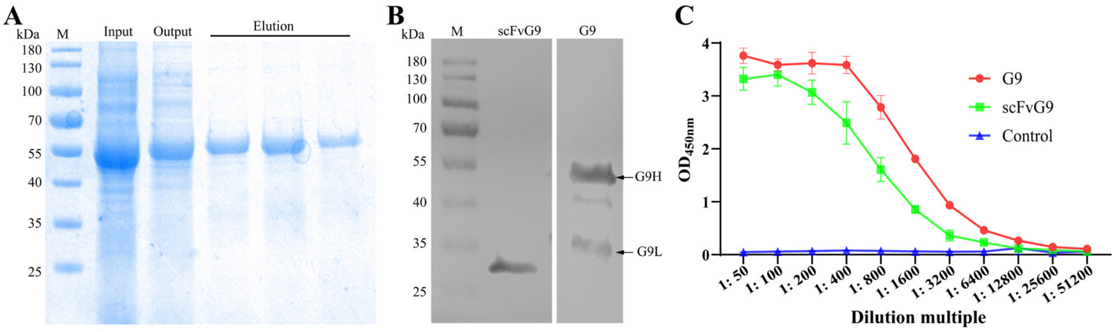

Fig. S5. Purification of GST-SWP1 fusion protein and measurement of scFvG9 titer. A: Recombinant GST-SWP1 fusion protein was purified from recombinant *E. coli* Rosetta. The SDS-PAGE showed recombinant GST-SWP1 fusion protein was eluted in elution buffer which contained 10 mM glutathione. B: G9 and scFvG9 were obtained by baculovirus expression system. The Western blot result confirmed the antibodies of G9 and scFvG9 fused with His tag were collected. C: The titer of G9 and scFvG9 was determined by indirect ELISA. ELISA plates were coated with recombinant GST-SWP1 fusion protein and incubated with G9 and scFvG9. Next, anti-His antibody （antibody from rabbit） was added and peroxidase-labeled goat anti-rabbit IgG was incubated. 3′,5,5′-tetramethylbenzidine (TMB) was used and stopped by 2 M H_2SO_4. Finally, the OD_{450nm}

value was measured, n=3.

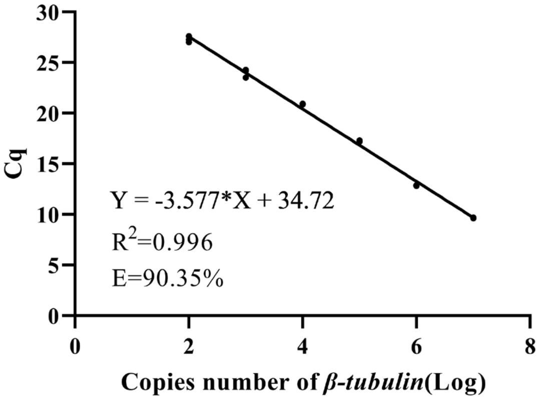

Fig. S6. The standard curve of *N. bombycis β-tubulin* was established for the analysis of pathogenic load. The standard template (the vector of pUC19-β-tubulin) was diluted 10-fold, and 1 μL of each diluted sample was used for qPCR. All samples were run in triplicate. The figures and linear regression analysis were generated with GraphPad Prism 8. According to the MIQE guidelines, PCR efficiency (E) = 10−1/slope – 1.

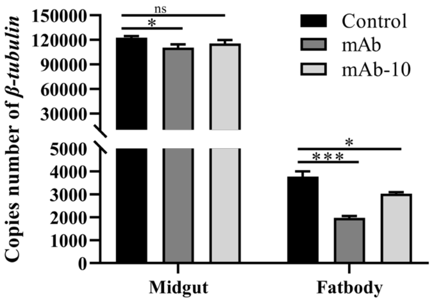

Fig. S7. The proliferation of *N. bombycis* was inhibited by antibody G9. Day 1 fifth instar *B. mori* naïve larvae were injected with monoclonal antibody G9 (mAb, 1 μg/larva), antibody G9 in dilutions of 1: 10 (mAb-10, 0.1 μg/larva) or negative serum (control, 1 μg/larva), and was then orally infected with *N. bombycis.* Midgut and fat body were collected at 6 days post-infection for extraction of the genome, which was used to evaluate the proliferation of microsporidia by qPCR. All samples were run in triplicate. The figures and statistics analysis were generated with GraphPad Prism 8. Bars represent the mean of three individual measurements ± SEM. Statistical significance was determined by an unpaired t-test, and statistically significant differences are represented with asterisks (*$p < 0.05$, *** $p < 0.001$).

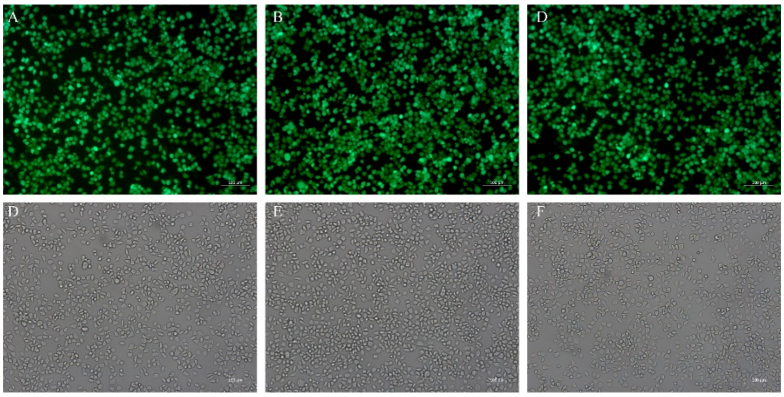

Fig. S8. Screening of transgenic cell lines. BmE cell lines of transfecting with G9-Neo (A, D), scFvG9 -Neo (B, E) and NscFvG9 -Neo (D, F) were selected by G418 for about 4 months.

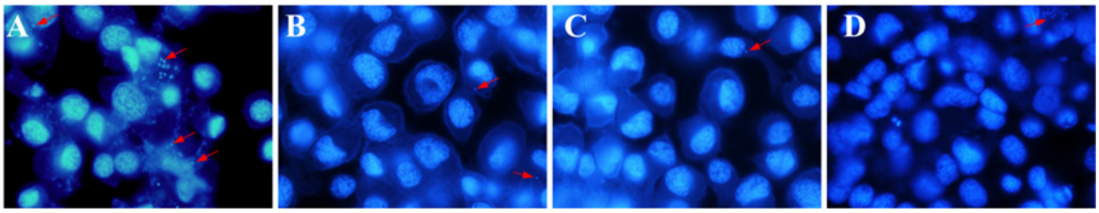

Fig. S9. Statistics of BmE cell lines infection rate. BmE cell lines transfecting with G9-Neo (B), scFvG9 -Neo (C) and NscFvG9 -Neo (D) were infected by *N. bombycis,* while BmE cells (A) were used as a control. After *N. bombycis* infection at 9 days, the infection rates were estimated by DAPI staining. The red arrow was the nucleus of microsporidia stained by DAPI and the cells containing *N. bombycis* were considered infected cells.

Table S1. The ELISA titer of monoclonal antibodies

Hybridoma lines	The supernatant of hybridoma cells	ascites
1F3	1：1.024×10^{3}	1：6.4×10^{5}
F10	1：2.56×10^{2}	1：6.4×10^{5}
G9	1：1.6×10^{3}	1：1.28×10^{6}

Table S2. Oligonucleotide primers

**Target gene and name**	**Sequence (5'→3')**
**rSWP1**	
>rSWP1-F	GATATC*ggatcc*ATGAATATTTTACTTGCTAC
>rSWP1-R	GC*aagctt*GAAAGGAATGGTATTGTCCAT
**rGST-SWP1**	
>rGST-SWP1-F	CCGCGT*ggatcc*ATGAATATTTTACTTGCTAC
>r GST-SWP1-R	CGGCCG*ctc gag*GAAAGGAATGGTATTGTCCAT
**G9**	
L-F	GAYATTGTGMTSACMCARWCTMCA
H-F	AGGTSMARCTGCAGSAGTCWGG
Oligo dT Primer	GCTGTCAACGATACGCTACGTAACGGCATGACAGTG$(T)_{18}$
3′ Racer Primer	GCTGTCAACGATACGCTACGTAACG
**G9H**	
>G9H-F1	GA*agatct*ATGGAAAGGCACTGGATCTTTCCCCTCCTGTTGTCAGTAAC
>G9H-F2	CCCCTCCTGTTGTCAGTAACTGCAGGTGTCCACTCCCAGGTCCAGCTGCAGTCT
>G9H-R	TTA*gcggccgc*TCATTAGTGGTGATGATGGTGATGTTTACCAGGAGAGTGGGAGA
**G9L**	
>G9L-F1	GA*agatct*ATGGAAAGGCACTGGATCTTTCCCCTCCTGTTGTCAGTAAC
>G9L-F2	CCCCTCCTGTTGTCAGTAACTGCAGGTGTCCACTCCGACATTGTGATCACCCAGTC
>G9L-R	TTA*gcggccgc*TCATTAGTGGTGATGATGGTGATGACACTCATTCCTGTTGAAGC
**scFv-G9**	
>scFv-G9H-F	GA*agatct*ATGGAAAGGCACTGGATCTTTC
>NscFv-G9H-F	GA*agatct*ATGCAGGTCCAGCTGCAGCAGTCT
>scFv-G9H-R	AGAGCCGCCACCACCGCTCCCACCACCACCTGAGGAGACGGTGACTGAGGT
>scFv-G9L-F	AGCGGTGGTGGCGGCTCTGGCGGCGGCGGATCAGACATTGTGATCACCCAGTCT
>scFv-G9L-R1	TTAGTGGTGATGATGGTGATGTGACCCTGAGCCTCCCCGTTTGATTTCCAGCTTGGC
>scFv-G9L-R2	TTA*gcggccgc*TCATTAGTGGTGATGATGGTGATGTGACCCTG

**Recombinant antibody**	
>rG9H-F	GGGCGC*ggatcc*ATGGAAAGGCACTGGATCT
>rG9H-R	TTA*gcggccgc*TCATTAGTGGTGATGATGGTGATGTTTACCAGGAGAGTGGGAGA
>rG9L-F	GA*cccggg*ATGGAAAGGCACTGGATCTTTCCCCTCCTGTTGTCAGTAAC
>rG9L-R	TTA*ggtacc*TCATTAGTGGTGATGATGGTGATGACACTCATTCCTGTTGAAGC
>rscFvG9-F	GGGCGC*ggatcc*ATGGAAAGGCACTGGATCT
>rscFvG9-R	TTA*gcggccgc*TCATTAGTGGTGATGATGGT
**β-tubulin**	
>β-tubulin-qF	AGAACCAGGAACAATGGACG
>β-tubulin-qR	AGCCCAATTATTACCAGCACC

Note: R=A,G; M=A,C; S=C,G; W=A,T; Y=C,T; N=A,C,G, T. Lower-case and italic letters represent restriction enzyme cleavage sites.
